# Microbial lipopolysaccharide‐induced inflammation contributes to cognitive impairment and white matter lesion progression in diet‐induced obese mice with chronic cerebral hypoperfusion

**DOI:** 10.1111/cns.14301

**Published:** 2023-06-08

**Authors:** Toshiki Inaba, Kazuo Yamashiro, Naohide Kurita, Yuji Ueno, Nobukazu Miyamoto, Kenichiro Hira, Sho Nakajima, Chikage Kijima, Ryohei Nakaguro, Takao Urabe, Nobutaka Hattori

**Affiliations:** ^1^ Department of Neurology Juntendo University School of Medicine Tokyo Japan; ^2^ Department of Neurology Juntendo University Urayasu Hospital Chiba Japan; ^3^ Department of Neurology University of Yamanashi Yamanashi Japan; ^4^ Metagen Inc. Yamagata Japan

**Keywords:** cognitive impairment, gut microbiota, lipopolysaccharide, neuroinflammation, white matter lesion

## Abstract

**Aims:**

White matter lesions (WMLs) are involved in the pathological processes leading to cognitive decline and dementia. We examined the mechanisms underlying the exacerbation of ischemia‐induced cognitive impairment and WMLs by diet‐induced obesity, including lipopolysaccharide (LPS)‐triggered neuroinflammation via toll‐like receptor (TLR) 4.

**Methods:**

Wild‐type (WT) and TLR4‐knockout (KO) C57BL/6 mice were fed a high‐fat diet (HFD) or low‐fat diet (LFD), and subjected to bilateral carotid artery stenosis (BCAS). Diet groups were compared for changes in gut microbiota, intestinal permeability, systemic inflammation, neuroinflammation, WML severity, and cognitive dysfunction.

**Results:**

In WT mice, HFD induced obesity and increased cognitive impairment and WML severity compared with LFD‐fed mice following BCAS. HFD caused gut dysbiosis and increased intestinal permeability, and plasma LPS and pro‐inflammatory cytokine concentrations. Furthermore, HFD‐fed mice had higher LPS levels and higher neuroinflammatory status, including increased TLR4 expression, in WMLs. In TLR4‐KO mice, HFD also caused obesity and gut dysbiosis but did not increase cognitive impairment or WML severity after BCAS. No difference was found between HFD‐ and LFD‐fed KO mice for LPS levels or inflammatory status in either plasma or WMLs.

**Conclusion:**

Inflammation triggered by LPS–TLR4 signaling may mediate obesity‐associated exacerbation of cognitive impairment and WMLs from brain ischemia.

## Graphical abstract

The present study showed that gut dysbiosis and ensuing lipopolysaccharide (LPS)‐induced neuroinflammation caused by a high‐fat diet can exacerbate cognitive impairments and white matter lesion progression after cerebral ischemia.

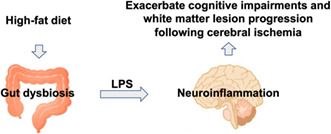

## 1 INTRODUCTION

White matter lesions (WMLs) mostly reflect demyelination and axonal loss as a consequence of chronic cerebral ischemia, leading to cognitive decline, and they are linked to dementia risk.^1^ The occurrence and severity of WMLs are strongly associated with systemic inflammation.^2^ Obesity is associated with systemic inflammation, which can lead to several diseases including type 2 diabetes mellitus, cardiovascular diseases, and neurodegenerative diseases.^3^, ^4^ Moreover, obesity in midlife increases the risk of dementia later in life^5^, ^6^ and is frequently associated with current WMLs.^7^ It has been suggested that systemic inflammation links obesity to WMLs.^8^

Recent evidence indicated that lipopolysaccharide (LPS) derived from gut microbiota may potentially trigger systemic inflammation in obese mice.^9^ Alteration of gut microbiota composition (gut dysbiosis) by a high‐fat diet (HFD) reportedly increased intestinal permeability, causing translocation of microbial LPS to the circulation and ensuing systemic inflammation, a condition known as metabolic endotoxemia.^10^ We previously reported that metabolic endotoxemia is associated with increased LPS in the brain and exacerbates neuroinflammation after stroke in genetically obese mice.^11^

Diet critically influences obesity and associated inflammatory status.^12^ Herein, we aimed to elucidate the mechanisms underlying obesity‐induced exacerbation of cognitive impairment and WMLs following ischemic injury. As the LPS–toll‐like receptor 4 (TLR4) signaling axis is a central regulator of systemic inflammation,^13^ we examined if the effects of diet‐induced obesity on ischemic injury are mitigated by TLR4 knockout (KO).

## 2 METHODS

### 2.1 Animal models

All animal experiments were approved by the Juntendo University Animal Ethics Committee (No. 1249) and performed in accordance with Animal Research: Reporting in Vivo Experiments (ARRIVE) guidelines for the care and use of laboratory animals. Five‐week‐old male wild‐type (WT) C57BL/6J mice (*n* = 64) were purchased from Charles River Japan (Kanagawa, Japan), and TLR4KO C57BL/6 mice (*n* = 40) from Oriental Bioservice (Kyoto, Japan). All mice were maintained on a 12‐h/12‐h light/dark cycle and administered a low‐fat diet (LFD) with 10.2 kcal% fat or an HFD with 56.7 kcal% fat (Japan CLEA Inc.) (Table S1) from 5 to 16 weeks of age. At 12 weeks of age, chronic cerebral hypoperfusion was induced via bilateral carotid artery stenosis (BCAS) using 0.18‐mm diameter microcoils (Sawane Spring Co. Ltd., Shizuoka, Japan) as described previously.^14^ All in vivo experiments and measurements were performed in a randomized and blinded manner.

### 2.2 Behavioral memory tests

Behavioral experiments were performed 14 and 28 days after BCAS. Spatial working memory was assessed using the *Y*‐maze test.^15^ Briefly, the Y‐maze consists of three arms, each 40 cm long, 13.5 cm high, and 4 cm wide. A mouse was placed at the end of the start arm and allowed to freely explore the maze while the sequence of arm entries was manually recorded. A mouse was considered to have entered an arm when all four paws were positioned in the arm runway. Consecutive entries into all three arms without re‐retries into previously explored arms were defined as an alternation. Spatial working memory was calculated as the ratio of actual alternations to maximum possible alternations (the total number of arm entries −2) × 100.

A novel object test was conducted to assess recognition memory.^16^ This test exploits the propensity of mice to explore novel objects over familiar objects. Briefly, mice were exposed individually to two identical objects for 10 min in the training trial. After a 1 h interval, mice were exposed to a copy of the familiar object from the training trial and a novel object for 5 min. The time spent exploring novel and familiar objects was recorded, and a discrimination ratio (novel object interaction/total interaction with both objects) was calculated as an index of recognition memory.

### 2.3 Microbial analysis

Fecal samples were collected from 12‐week‐old mice fed either HFD or LFD for microbial analysis. Details on microbial DNA extraction, amplification, and bioinformatics are found in Appendix S1.

### 2.4 Plasma measurements

Plasma glucose concentration was measured using a blood glucose meter (Johnson & Johnson) and plasma LPS concentration using a kit based on a Limulus amebocyte extract (LPS kit, Cusabio). Plasma interleukin (IL)‐6 and IL‐1β were measured using enzyme‐linked immunosorbent assays (ELISAs, R&D Systems) with sensitivities of 1.6 pg/mL and 2.31 pg/mL, respectively.

### 2.5 Intestinal permeability

Intestinal permeability was measured as described previously.^11^ Briefly, mice were fasted for 6 h and administered 4000‐kDa fluorescein isothiocyanate (FITC)‐dextran (440 mg/kg body weight, 100 mg/mL) dissolved in deionized water by oral gavage. Mice were anesthetized 1 h after oral gavage, and a cardiac puncture was performed to collect blood. Plasma samples were diluted in equal volumes of phosphate‐buffered saline (PBS) and analyzed for FITC‐dextran content using a fluorescence spectrophotometer (Mithras^2^ LB 943 microplate reader, Berthold Technologies).

### 2.6 Brain tissue preparation

Mice were deeply anesthetized by intraperitoneal injection of pentobarbital (50 mg/kg) and transcardially perfused with 20 mL of ice‐cold PBS to remove blood from brain capillaries. For histological analysis, brains were subsequently perfused with 4% paraformaldehyde in PBS, rapidly removed, post‐fixed in the same solution overnight at 4°C, and cryopreserved by immersion in 30% sucrose for at least 24 h. Brains were then frozen, and 20‐μm‐thick consecutive coronal sections were prepared on a cryostat (CM 1900; Leica Instruments, Nusslosh, Germany). Coronal sections between +0.86 mm and + 0.50 mm from the bregma were used for myelin staining, IgG staining, and immunohistochemistry. For immunoblotting and ELISA, brains were removed immediately after PBS perfusion without fixation. The corpus callosum was separated using sharp forceps under a microscope and sectioned into three 1‐mm coronal sections starting from 2 mm behind the end of the olfactory bulb. All sections were stored at −80°C until analysis.

### 2.7 Myelin staining

The corpus callosum was evaluated for WMLs by Kluver‐Barrera staining of three coronal sections per animal. The severity of WMLs was graded as 0 (normal), 1 (disarrangement of nerve fibers), 2 (formation of marked vacuoles), or 3 (disappearance of myelinated fibers) according to a previous report.^17^ In addition, three coronal sections per animal were incubated with FluoroMyelin Green Fluorescent Myelin Stain (1:300; Molecular Probes). Images were quantified using ImageJ software (National Institutes of Health; https://imagej.nih.gov/ij/).

### 2.8 IgG staining

To evaluate the permeability of the blood–brain barrier (BBB), sections were incubated in 3% H_2_O_2_, blocked with 10% bovine serum albumin (Sigma‐Aldrich) in PBS, and incubated overnight at 4°C with donkey anti‐mouse IgG (1:300; Vector Laboratories). Immunoreactivity was visualized using the avidin‐biotin complex method (Vectastain ABC kit, Vector Laboratories) and quantified using ImageJ.

### 2.9 Immunohistochemistry for astrocyte, microglial, and oxidative stress markers

Immunohistochemistry was performed on free‐floating coronal sections. For single staining, sections were incubated with one of the following primary antibodies: rabbit polyclonal antibody against ionized calcium‐binding adaptor molecule (Iba‐1; 1:500; Wako Pure Chemical Industries), rabbit polyclonal antibody against glial fibrillary acidic protein (GFAP; 1:500; Cosmo Bio Co.), rabbit polyclonal antibody against single‐stranded DNA (ssDNA; 1:50; IBL), rabbit polyclonal antibody against 4‐hydroxy‐2‐nonenal (4‐HNE; 1:50; JalCA), or mouse monoclonal antibody against 8‐hydroxy‐deoxyguanosine (8‐OHdG; 1:50; JalCA). The sections were then incubated sequentially with secondary antibodies and avidin‐biotin‐peroxide complex reagent (Vector Laboratories). For double‐immunofluorescence staining, sections were incubated with primary antibodies against Iba‐1 (1:500; Wako Pure Chemical Industries) and transmembrane protein 119 (TMEM119; 1:150; Abcam), followed by incubation with Cy™3‐conjugated secondary antibody (1:1000; Jackson ImmunoResearch) for visualization of Iba‐1 and with FITC‐conjugated secondary antibody (1:1000; Jackson ImmunoResearch) for visualization of TMEM119. Immunostained sections were covered with Vectashield mounting medium (Vector Laboratories) and immunofluorescence images were obtained using a Zeiss LSM 780 confocal microscope (Carl Zeiss). The number of immunopositive cells was counted by a researcher blinded to the experimental conditions. The average number of immunopositive cells per mouse was calculated as the sum of immunopositive cells in all stained sections from the same mouse.

### 2.10 Immunoblotting

Proteins were extracted from frozen corpus callosum tissue by homogenization in lysis buffer containing a protease inhibitor cocktail, followed by centrifugation (14,000 × g for 10 min at 4°C) to remove debris. An equal amount of protein from each sample was loaded onto sodium dodecyl sulfate‐polyacrylamide gels, separated by electrophoresis, and transferred to polyvinylidene difluoride membranes. The membranes were blocked in Block Ace (Dainichi‐Seiyaku) and then incubated overnight at 4°C with antibodies against myelin basic protein (MBP; 1:5000; Abcam), LPS (1:5000; Abcam), TLR4 (1:5000; Santa Cruz Biotechnology), occludin (1:5000; Abcam), or α‐tubulin (1:10000; Santa Cruz Biotechnology), followed by incubation with peroxidase‐conjugated secondary antibodies (1:5000; Santa Cruz Biotechnology). Immunolabeling was visualized using an enhanced chemiluminescence reagent (Amersham Biosciences) and quantified by densitometry using ImageJ.

### 2.11 Cytokine measurement in white matter

The frozen corpus callosum was homogenized in PBS containing a protease inhibitor cocktail and centrifuged at 14,000 × g for 10 min at 4°C. The supernatant was collected and concentrations of IL‐6 and IL‐1β were measured immediately by ELISA kits (R&D Systems). Cytokine concentrations in the corpus callosum are expressed as picograms per milligram of protein.

### 2.12 Statistical analysis

Samples sizes sufficient for detecting 30%–50% differences between in vivo models were estimated for *α* = 0.05 and *β* = 0.8. Results are presented as mean ± SD if normally distributed or median ± interquartile range if nonnormally distributed according to the Kolmogorov–Smirnov test. Group data were compared using Student's *t*‐test when normally distributed or Mann–Whitney *U*‐test when nonnormally distributed. Statistical analyses were performed using GraphPad Prism version 8.4.3 (GraphPad Software). A *p <* 0.05 (two‐tailed) was considered statistically significant for all tests.

## 3 RESULTS

### 3.1 Obesity from high‐fat diet (HFD) feeding exacerbated cognitive impairment and WMLs induced by cerebral hypoperfusion in WT mice

**FIGURE 1:**
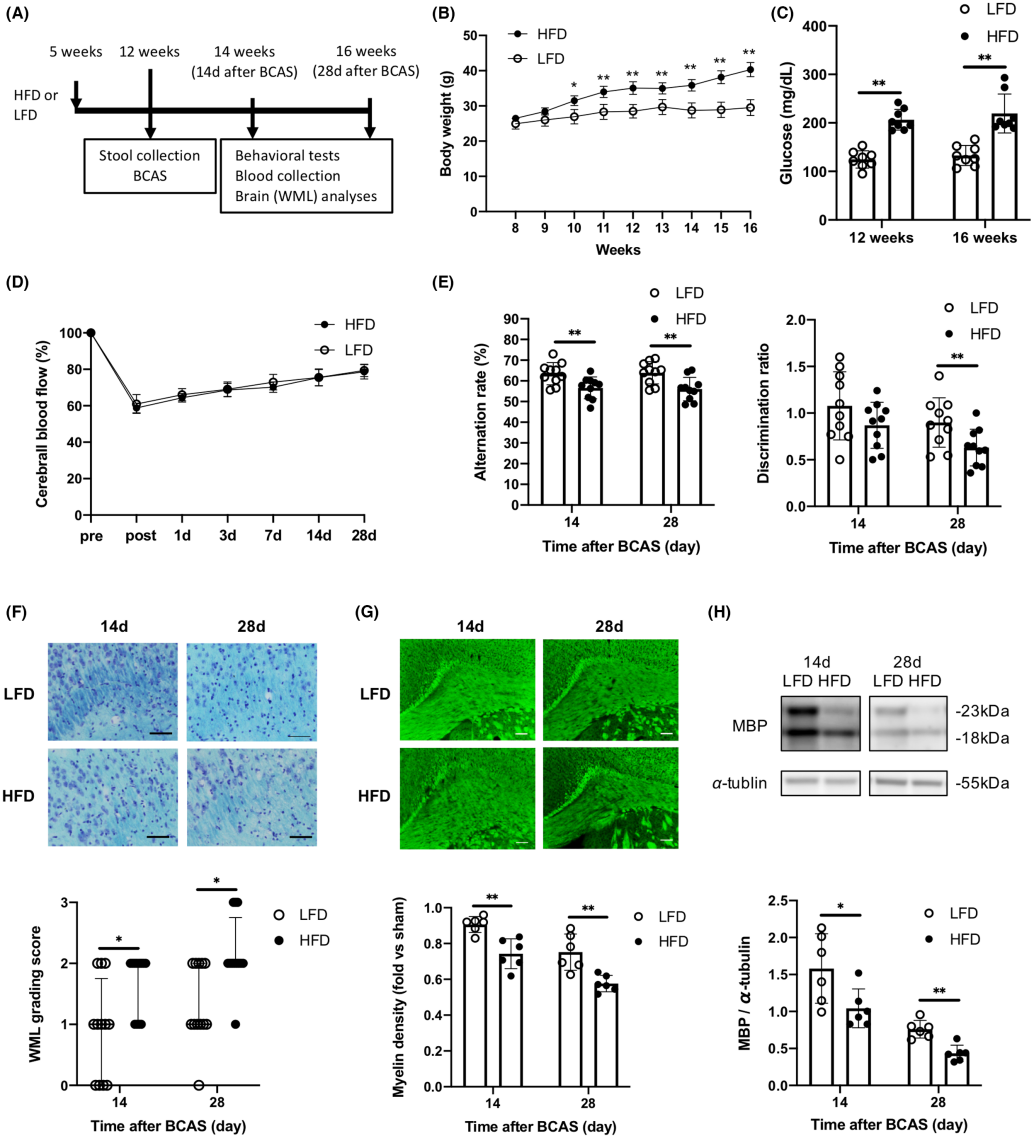
High‐fat diet (HFD) feeding alters metabolic parameters, impairs cognitive function, and increases white matter lesion (WML) burden in wild‐type mice. (A) Experimental design, (B) body weight (*n* = 8 per group), (C) plasma glucose levels (*n* = 8 per group), (D) cerebral blood flow before (pre); immediately after (post); and 1, 3, 7, 14, and 28 days after bilateral carotid artery stenosis (*n* = 8 per group). (E) Results of the Y‐maze test for spatial working memory (left) and novel object test for recognition memory (right) (*n* = 10 per group). (F) Kluver‐Barrera staining in the corpus callosum and WML grading score (*n* = 10 per group). Scale bar: 50 μm. (G) FluoroMyelin staining and relative FluoroMyelin intensity (*n* = 6 per group). Scale bar: 100 μm. (H) Western blotting and densitometric analysis of myelin basic protein expression in the corpus callosum (*n* = 6 per group). Results are presented as mean ± SD (B–E, G, H) and median ± interquartile range (F). *p < 0.05, **p < 0.01.

WT mice were fed either an LFD or an HFD from 5 weeks of age (Figure 1A). Mean body weight was significantly greater in the HFD‐fed mouse group (HFD mice) than in the LFD‐fed mouse group (LFD mice) from 10 weeks of age (Figure 1B). In addition, blood glucose levels were higher in HFD mice at 12 and 16 weeks of age (Figure 1C). Both groups were subjected to experimental BCAS at 12 weeks of age (Figure 1A), which immediately reduced cerebral blood flow (CBF) by approximately 40% without a group difference (Figure 1D). However, spatial memory as assessed by alternating arm exploration in the Y‐maze test was more severely impaired in HFD mice at 14 and 28 days after BCAS (Figure 1E). Further, HFD mice exhibited poorer recognition memory in the novel object test than LFD mice at 28 days after BCAS (Figure 1E). In the corpus callosum, WMLs were also more severe in HFD mice than LFD mice according to WML grade (Figure 1F), myelin staining (Figure 1G), and MBP expression on western blots (Figure 1H).

### 3.2 HFD caused gut dysbiosis and increased intestinal permeability, plasma LPS, and plasma pro‐inflammatory cytokines in WT mice

**FIGURE 2:**
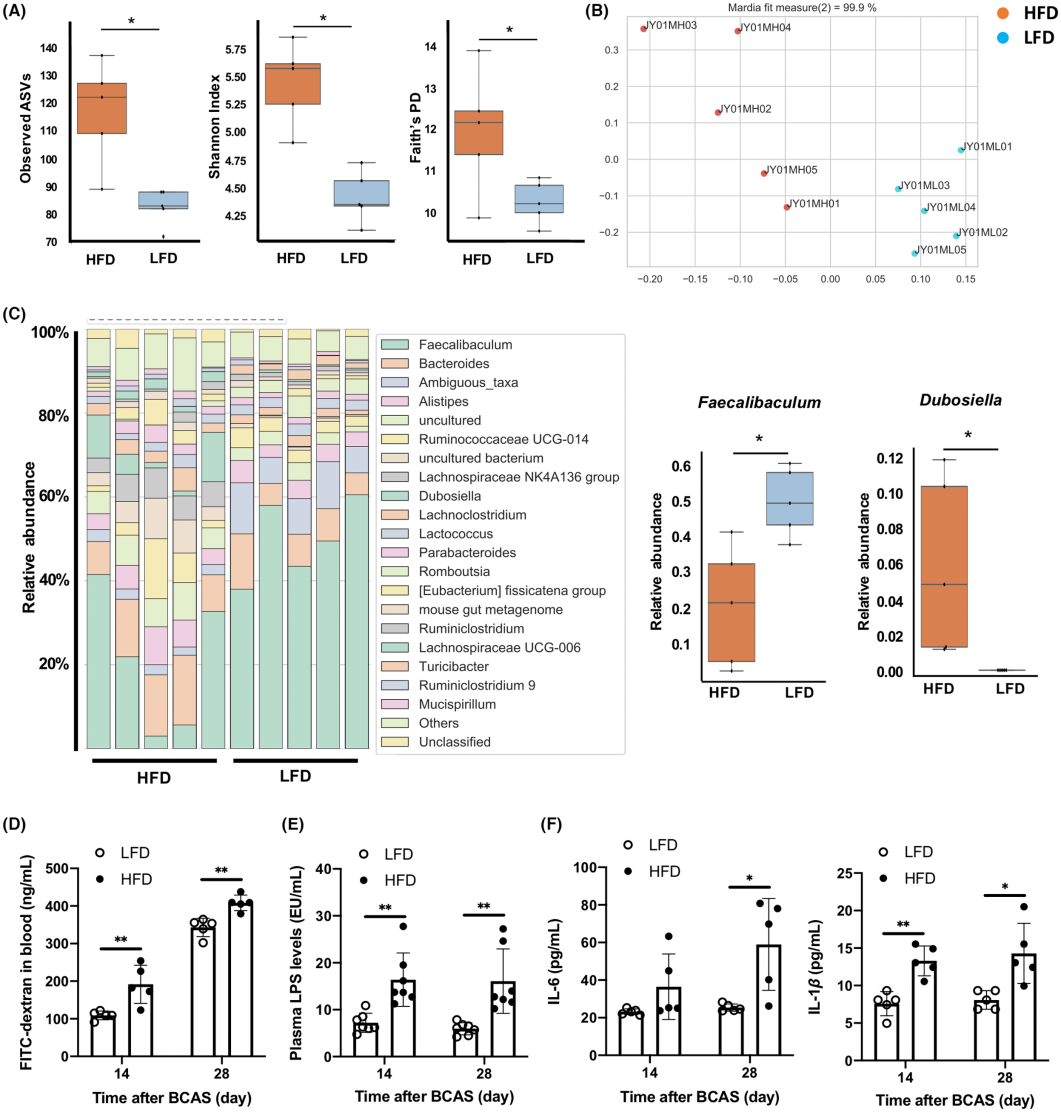
High‐fat diet (HFD) feeding alters gut microbiota composition and increases intestinal permeability, plasma lipopolysaccharide (LPS) concentration, and plasma pro‐inflammatory cytokine concentrations in wild‐type mice. (A) Alpha diversity metrics for HFD and low‐fat diet mice, including richness (left), Shannon index (center), and Faith's phylogenetic diversity (right) (*n* = 5 per group). (B) Principal coordinate analysis based on weighted UniFrac distances (*n* = 5 per group). (C) Relative abundance of bacteria at the genus level (*n* = 5 per group). (D) Intestinal permeability (*n* = 5 per group). (E) Plasma LPS concentrations (*n* = 7 per group). (F) Plasma concentrations of IL‐6 and IL‐1β (*n* = 5 per group). Results are presented as median ± interquartile range (A, C) and mean ± SD (D–F). *p < 0.05, **p < 0.01.

The impact of diet on microbiota composition was evaluated by 16S rRNA microbiome analysis of fecal samples collected from 12‐week‐old WT mice maintained for 7 weeks on the HFD or LFD. The HFD group exhibited higher bacterial diversity than the LFD group (Figure 2A), and principal coordinate analysis showed that samples were clustered according to diet (Figure 2B). The relative abundance of *Faecalibaculum* was lower in HFD mice, whereas the relative abundance of *Dubosiella* was higher in HFD mice than LFD mice (Figure 2C). Moreover, intestinal permeability and plasma LPS levels were significantly greater among HFD mice than LFD mice at 14 and 28 days after BCAS (Figure 2D,E). The plasma levels of pro‐inflammatory cytokines IL‐6 and IL‐1β were also higher in HFD mice (Figure 2F).

### 3.3 Obesity from HFD feeding exacerbated BBB impairment, white matter LPS accumulation, and WML progression in WT mice following BCAS

**FIGURE 3:**
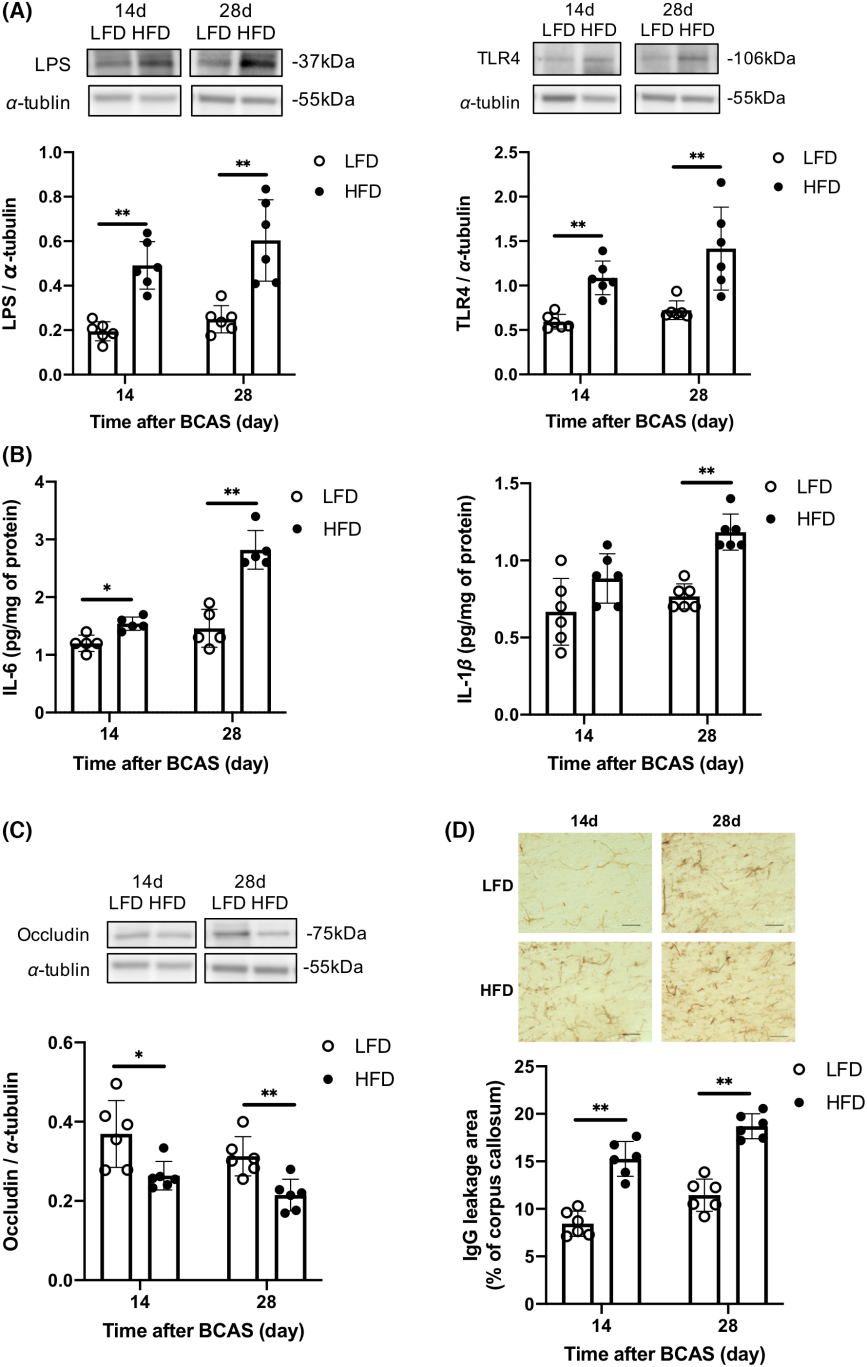
High‐fat diet feeding increases lipopolysaccharide (LPS) concentration, inflammatory cytokine concentrations, TLR4 expression, and blood–brain barrier (BBB) permeability in the corpus callosum of wild‐type mice 14 and 28 days after bilateral carotid artery stenosis. (A) Western blotting and densitometric analyses of LPS and TLR4 (*n* = 6 per group). (B) IL‐6 (*n* = 5 per group) and IL‐1β levels (*n* = 6 per group). (C) Western blotting and densitometric analysis of occludin (*n* = 6 per group). (D) BBB permeability as assessed by IgG (*n* = 6 per group). Scale bar: 50 μm. Results are presented as mean ± SD. *p < 0.05, **p < 0.01.

LPS levels and TLR4 expression were significantly higher in the corpus callosum of HFD mice than LFD mice at 14 and 28 days after BCAS (Figure 3A). In addition, IL‐6 and IL‐1β concentrations were higher in the corpus callosum of HFD mice (Figure 3B). Conversely, the tight junction protein occludin was downregulated in HFD mice (Figure 3C), and BBB permeability was significantly higher in the corpus callosum of HFD mice than LFD mice as measured by IgG staining (Figure 3D).

**FIGURE 4:**
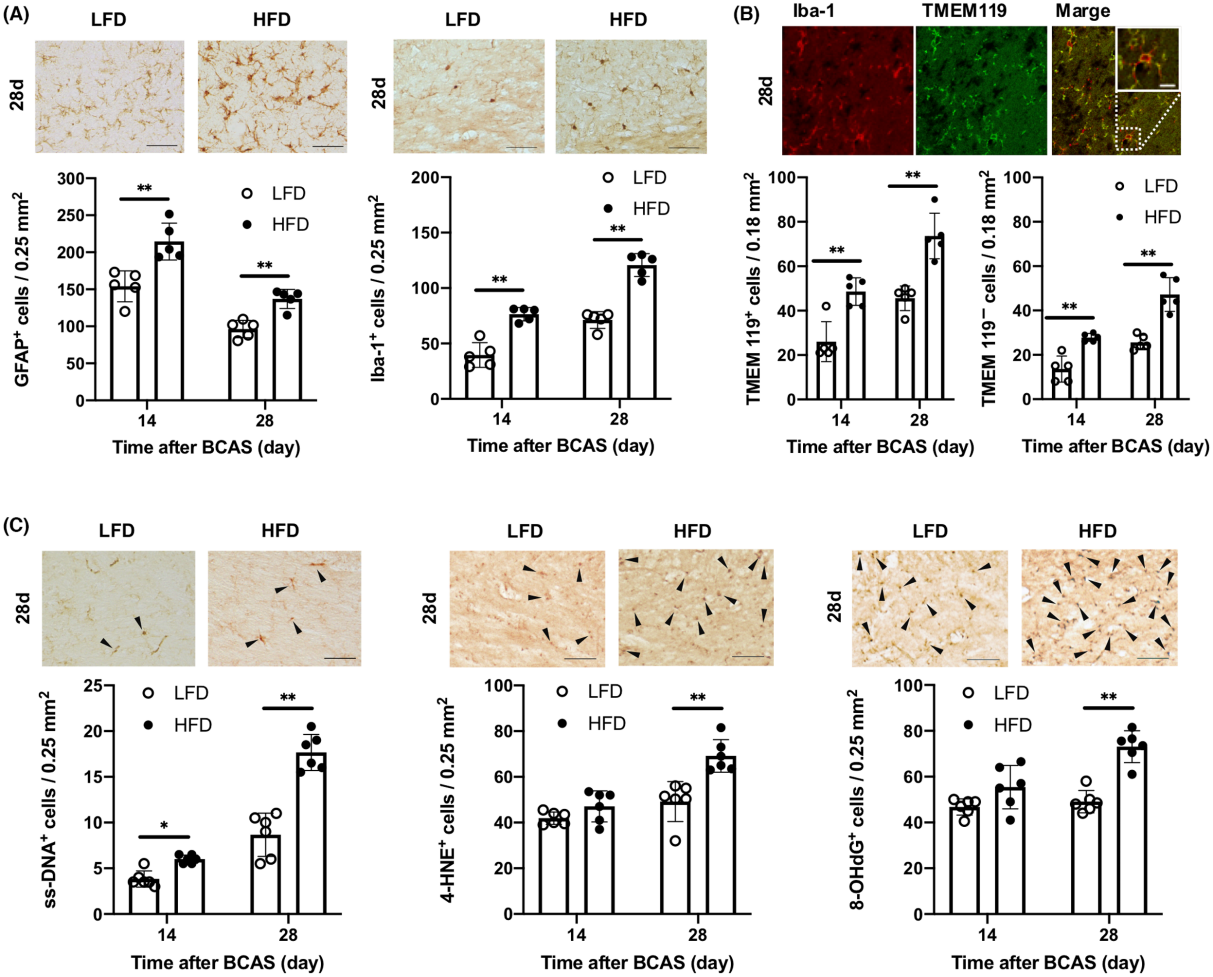
High‐fat diet feeding alters glial cell phenotypes and increases expression of oxidative stress markers in the corpus callosum of wild‐type mice 14 and 28 days after bilateral carotid artery stenosis. (A) Representative images and cell counts of astrocytes (glial fibrillary acidic protein‐positive cells) and microglia/macrophages (Iba‐1‐positive cells) in the corpus callosum (*n* = 5 per group). Scale bar: 50 μm. (B) Representative double‐immunofluorescence images of Iba‐1 and TMEM119 and cell counts of TMEM119‐positive (left) and TMEM119‐negative cells (right) among Iba‐1‐positive cells (microglia/macrophages) in the corpus callosum (*n* = 5 per group). Scale bar: 10 μm. (C) Representative images and counts of cells positive for oxidative stress markers (ssDNA, 4‐HNE, 8‐OHdG) (arrowheads) in the corpus callosum (*n* = 6 per group). Scale bar: 50 μm. Results are presented as mean ± SD. *p < 0.05, **p < 0.01.

The numbers of GFAP‐positive (reactive) astrocytes and Iba‐1‐positive (activated) microglia/macrophages were also higher in the WMLs of HFD mice than LFD mice at 14 and 28 days after BCAS (Figure 4A). Furthermore, the number of Iba‐1‐positive cells expressing TMEM119, a specific marker of tissue‐resident microglia, was higher in the WMLs of HFD mice, as was the number of Iba‐1‐positive cells not expressing TMEM119 (Figure 4B). Oxidative stress was also elevated in the corpus callosum of HFD mice compared to LFD mice as evidenced by a greater number of cells positive for the oxidative stress markers ssDNA, 4‐HNE, and 8‐OHdG (Figure 4C).

### 3.4 HFD did not promote cognitive impairment and WMLs in TLR4KO mice

**FIGURE 5:**
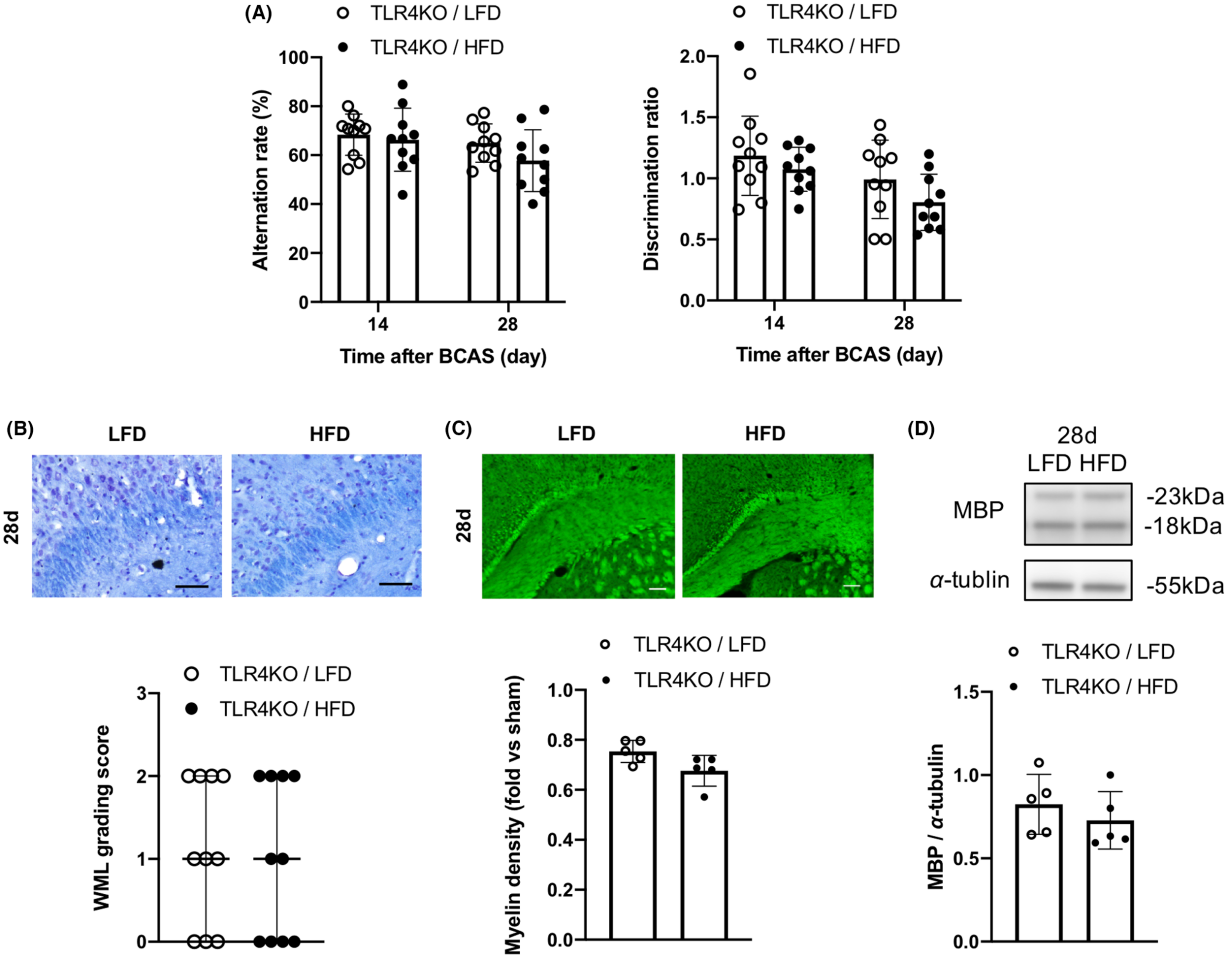
High‐fat diet feeding does not markedly impair cognitive function or white matter lestions (WML) burden in TLR4KO mice. (A) Results of the spatial memory test (left) and recognition memory test (right) 14 and 28 days after bilateral carotid artery stenosis (*n* = 10 per group). (B) Kluver‐Barrera staining of the corpus callosum and WML grading scores (*n* = 10 per group). Scale bar, 50 μm. (C) FluoroMyelin staining and relative FluoroMyelin intensity (*n* = 5 per group). Scale bar: 100 μm. (D) Western blotting and densitometric analysis of myelin basic protein (*n* = 5 per group). Results are presented as mean ± SD (A, C, D) and median ± interquartile range (B).

The experiments detailed in Sections 3.1 to 3.3 were conducted simultaneously in TLR4KO mice (Figure 1A). Similar to HFD‐fed WT mice, mean body weight was significantly higher among HFD‐fed TLR4KO mice compared with LFD‐fed TLR4KO mice from 11 weeks of age (Figure S1A). In addition, blood glucose concentrations were higher in HFD‐fed KO mice than LFD‐fed KO mice at 12 and 16 weeks of age (Figure S1B). Also in accord with findings from WT mice, the CBF reduction was similar in both diet groups of KO mice after BCAS (Figure S1C). However, unlike the WT diet groups, spatial memory, and recognition memory 14 and 28 days after BCAS did not differ between HFD‐fed and LFD‐fed TLR4KO mice (Figure 5A). Further, white matter damage in the corpus callosum as assessed by histological grading (Figure 5B), myelin staining (Figure 5C), and MBP expression on western blots (Figure 5D) did not differ between diet groups 28 days after BCAS.

### 3.5 HFD caused gut dysbiosis but did not increase LPS levels in plasma or WMLs of TLR4KO mice

**FIGURE 6:**
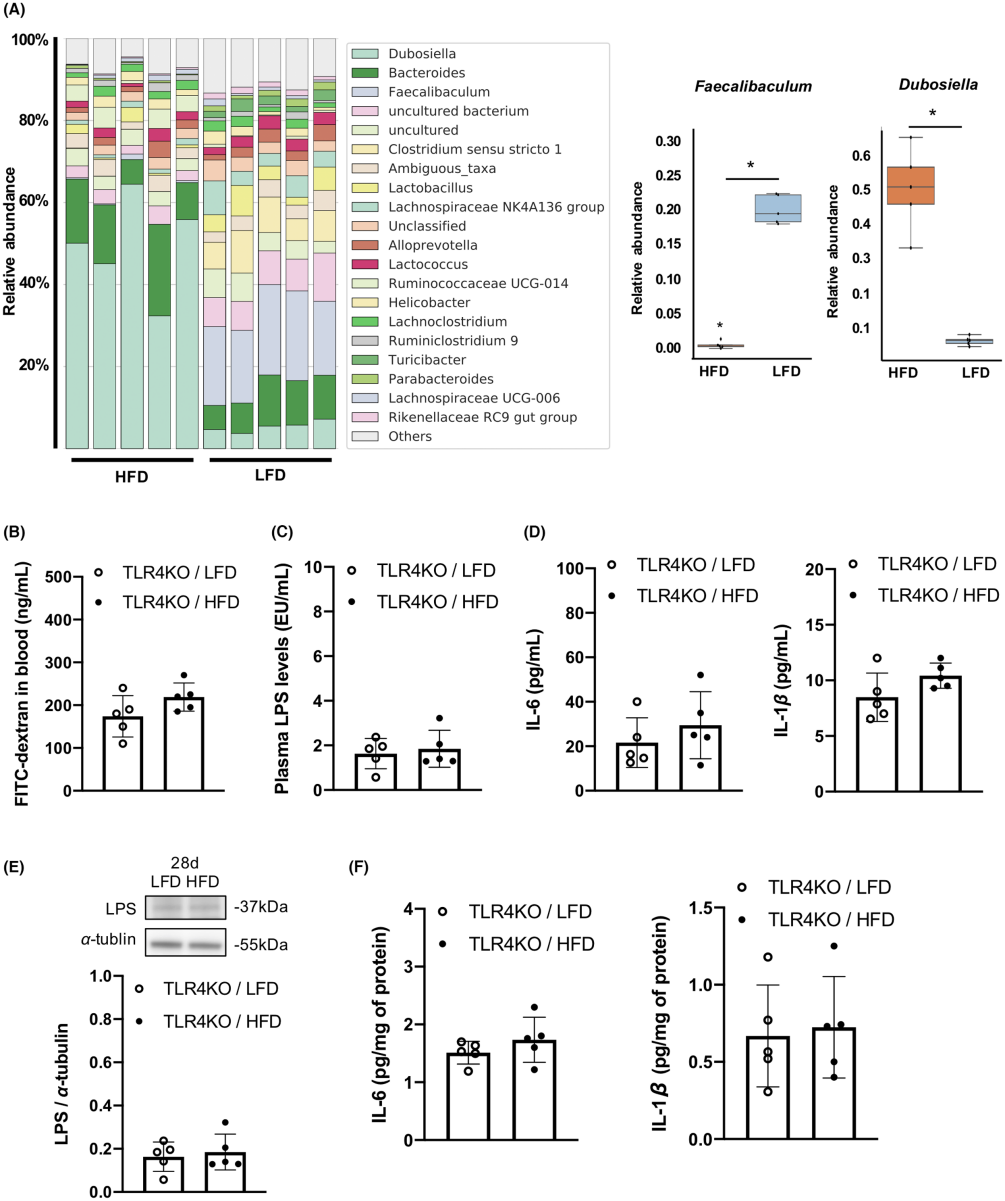
High‐fat diet (HFD) feeding alters gut microbial composition but not intestinal permeability or the concentrations of lipopolysaccharide (LPS) and inflammatory cytokines in plasma and corpus callosum of TLR4KO mice after BCAS. (A) Relative abundance of bacteria at the genus level (*n* = 5 per group). (B) Intestinal permeability (*n* = 5 per group). (C) Plasma LPS concentration 28 days after bilateral carotid artery stenosis (BCAS) (*n* = 5 per group). (D) Plasma IL‐6 and IL‐1β concentrations 28 days after BCAS (*n* = 5 per group). (E) Western blotting and densitometric analysis of LPS (*n* = 5 per group), and (F) IL‐6 and IL‐1β in the corpus callosum 28 days after BCAS (*n* = 5 per group). Results are presented as median ± interquartile range (A) and mean ± SD (B–F). *p < 0.05.

Fecal samples were collected from 12‐week‐old TLR4KO mice maintained for 7 weeks on the HFD or LFD to investigate microbial composition. Bacterial diversity was lower in HFD‐fed KO mice than LFD‐fed KO mice (Figure S2A) and principal coordinate analysis showed that samples were clustered according to diet (Figure S2B). The relative abundance of *Faecalibaculum* was lower in HFD‐fed KO mice, whereas the relative abundance of *Dubosiella* was higher in HFD‐fed KO mice than LFD‐fed KO mice (Figure 6A). In contrast, intestinal permeability and plasma concentrations of LPS and pro‐inflammatory cytokines did not differ between HFD‐ and LFD‐fed KO mice (Figure 6B–D). Also distinct from findings in WT mice, LPS levels and pro‐inflammatory cytokine concentrations (Figure 6E,F), expression levels of occludin, BBB permeability, and the numbers of cells positive for GFAP and Iba‐1 (Figure S3A–C) in the corpus callosum did not differ between HFD‐ and LFD‐fed KO mice.

## 4 DISCUSSION

The present study demonstrates that an HFD and concomitant obesity can exacerbate spatial and recognition memory impairments and WMLs induced by BCAS in mice. HFD‐fed WT mice developed gut dysbiosis and exhibited greater intestinal permeability and plasma LPS and pro‐inflammatory cytokine concentrations compared with LFD‐fed lean mice. Furthermore, HFD‐fed mice had higher LPS levels and higher neuroinflammatory status, including increased TLR4 expression, in WMLs. Although HFD also caused obesity and gut dysbiosis in TLR4KO mice, cognitive impairment and WMLs were less severe, and no difference was found between HFD‐ and LFD‐fed KO mice for LPS levels or inflammatory status in either plasma or WMLs. These findings suggest that gut microbial LPS‐induced inflammation contributes to cognitive impairment and WML progression in obese mice and that these effects are dependent on TLR4 signaling.

Gut microbial LPS has been implicated in the pathogenesis of several obesity‐associated disorders including type 2 diabetes mellitus,^10^ stroke,^11^ and cardiovascular disease.^18^ As in mouse models, obese phenotypes are associated with elevated circulating LPS levels in humans.^19^, ^20^ Gut dysbiosis reportedly increased intestinal permeability, leading to the translocation of LPS from the intestinal lumen into the circulation and the triggering of systemic inflammation, in both genetic models of obesity^11^, ^21^ and HFD‐induced obese mice.^22^, ^23^ Consistent with these previous studies, HFD increased intestinal permeability and the plasma concentrations of LPS, IL‐6, and IL‐1β in WT mice. Additionally, HFD‐induced dysbiosis led to a decreased relative abundance of *Faecalibaculum* and an increased relative abundance of *Dubosiella*, two of the major genera detected in the present study. Reduction in *Faecalibaculum* abundance was consistent with a previous study of HFD‐fed mice.^24^ *Faecalibaculum* are beneficial gut bacteria as they produce short‐chain fatty acids that protect against intestinal tumor growth, reduce inflammation, and stabilize the intestinal epithelial barrier.^25^, ^26^ Conversely, increased *Dubosiella* abundance was associated with intestinal inflammation in HFD‐induced obese mice.^27^ Therefore, gut dysbiosis characterized by lower *Faecalibaculum* abundance and higher *Dubosiella* abundance may cause pro‐inflammatory gut milieu, potentially leading to intestinal barrier dysfunction, LPS translocation, and systemic inflammation.

Microbial diversity was greater in HFD‐fed obese mice than LFD‐fed lean mice. Conversely, previous studies reported that obesity reduced decreased microbial diversity.^28^, ^29^ Microbial diversity can be affected by dietary fiber.^24^ Higher fiber content in the HFD‐fed mice compared with the LFD‐fed (5.5% and 4.0% cellulose, respectively) mice may have increased the microbial diversity in the HFD‐fed mice. However, greater fiber intake (15%–25% fiber) is thought to be needed to reverse the adverse effects of HFD on gut microbiota.^24^ Therefore, despite the lack of clarity, the minor difference in dietary fiber may not have contributed to the remarkable intergroup differences in microbiota profile, inflammation, white matter damage, and cognition.

The BBB maintains metabolic and immune homeostasis in the brain by selectively regulating or blocking the passage of various molecules and cells from circulation. Loss of BBB integrity enables neurotoxic molecules, blood‐born cells including immune cells, and various pathogens to enter the brain and trigger inflammatory responses, which may activate multiple neurodegenerative pathways.^30^ Lipopolysaccharide‐induced systemic inflammation can disrupt BBB function by damaging structural constituents including neurovascular endothelial cells, tight junctions connecting these cells, glycocalyx surrounding various neurovascular and neural cells, and astrocytic processes abutting the neurovasculature (endfeet).^31^ In the present study, the HFD exacerbated BBB disruption following BCAS as evidenced by increased IgG staining and decreased expression of the tight junction protein occludin, resulting in LPS accumulation, inflammatory cell infiltration, resident immune cell activation, and ultimately progression of WMLs.

LPS accumulation in WMLs was associated with enhanced local expression of TLR4 and the pro‐inflammatory cytokines IL‐6 and IL‐1β. Further, the number of Iba‐1‐positive microglia/macrophages was higher in WMLs of HFD‐fed than LFD‐fed WT mice. Microglia are the primary drivers of early neuroinflammation and are strongly implicated in the pathogenesis of cognitive impairment and neurodegenerative disorders including Alzheimer's disease.^32^ TLR4 is primarily expressed on microglia in the central nervous system (CNS), and oligodendroglial injury via activation of the innate immune response in microglia may be involved in the cellular mechanisms of LPS‐induced WMLs.^33^ Moreover, systemic LPS administration increased inflammatory cytokine expression and promoted inflammatory monocyte migration from the periphery into the brain.^34^ In the current study, both microglia positive for TMEM119, a specific marker of tissue‐resident microglia,^35^ and microglia negative for TMEM119 were more abundant in the WMLs of HFD‐fed mice than LFD‐fed mice, suggesting that elevated LPS caused infiltration of activated macrophages and activation of resident microglia, leading to neuroinflammation and WML progression. The WMLs of HFD‐fed mice also contained a greater number of cells positive for the oxidative stress markers ssDNA, 4‐HNE, and 8‐OHdG. Activated microglia are the primary cellular source of oxidative products that promote neuroinflammation and neurodegeneration.^36^ Additionally, oxidative stress interferes with white matter repair by disrupting oligodendrocyte precursor cell renewal.^37^

The WMLs in HFD‐fed mice contained a greater number of cell expressing GFAP, a marker for reactive astrocytes. Reactive astrocytes have been classified into two subtypes with distinct effects on neuronal survival during inflammation, neurotoxic A1 and neuroprotective A2.^38^ Although both types lack TLR4, microglia activated by LPS stimulation selectively induce A1 astrocytes, which contributed to the death of neurons and oligodendrocytes.^38^ Indeed, we previously reported that A1‐like astrocyte numbers were increased in the corpus callosum after BCAS and that these cells contributed to WML progression by impairing oligodendrocyte differentiation.^14^

Evidently, gut microbiota is intimately involved in CNS disorders^39^, ^40^ and that microbial LPS is a critical mediator of these pathogenic effects.^41^, ^42^ LPS concentrations are reportedly higher in the serum and cerebrospinal fluid of Alzheimer's disease patients^41^ and in the postmortem brains of Alzheimer's disease patients compared to age‐matched controls.^42^ Increased plasma concentrations of LPS and soluble CD14, both markers of gut dysbiosis and compromised intestinal integrity, were identified in healthy individuals showing cognitive decline,^43^ whereas in a recent hospital‐based cohort study, higher plasma LPS concentration indicated cognitive decline and cerebral small vessel disease, including WMLs, and was correlated with low fecal concentrations of beneficial microbial metabolites including lactic and acetic acid.^44^

Moreover, gut dysbiosis and LPS–TLR4 signaling have been implicated in intestinal inflammation and increased intestinal permeability among HFD‐fed obese mice.^22^ In the present study, HFD‐fed TLR4KO mice showed distinct changes in the abundance of two major bacterial genera, *Faecalibaculum* and *Dubosiella*, similar to HFD‐fed WT mice. However, the precise effects of HFD feeding on microbial diversity were different between WT and TLR4KO mice. In addition to diet, host factors including TLRs affect the modulation of gut microbiota composition.^45^ Consistent with previous studies,^22^ HFD did not increase intestinal permeability and plasma LPS and pro‐inflammatory cytokine levels in TLR4KO mice. In addition, HFD did not increase LPS accumulation and the neuroinflammatory status within WMLs following BCAS. These findings suggest that gut microbial LPS may link systemic inflammation to cognitive impairment and WMLs via TLR4. However, other TLR4 ligands such as saturated fatty acids may be involved in HFD‐induced inflammation.^46^

The diets used in the present study are similar to those used previously to induce obesity or maintain lean body mass in rodents.^47^ In the current study, fat accounted for 56.7% of total energy (in kcal) in the HFD and 10.2% of total energy in the LFD, whereas obesity was induced in previous studies using HFDs in which 45%–60% of total caloric content was derived from fat and sustained lean weight using LFDs in which approximately 10% of energy was derived from fat.^47^ Rodents are useful models to elucidate obesity physiology; however, human and animal studies show differences in defining HFD and LFD. In humans, the typical Western‐style HFD derives 30%–40% of total energy from fat,^48^ whereas diets with fat content not exceeding 30% of total energy are considered low in fat.^49^

## 5 CONCLUSION

Gut dysbiosis and ensuing LPS‐induced inflammation due to an HFD can exacerbate cognitive impairment and WML progression following cerebral ischemia.

## AUTHOR CONTRIBUTIONS

TI, KY, and TU designed the experiments. TI, NK, YU, NM, KH, SN, CK, and RN performed the experiments and analyzed the data. TI and KY drafted the manuscript. KY, TU, and NH revised the manuscript. All authors read and approved the final manuscript.

## CONFLICT OF INTEREST STATEMENT

The authors declared that they have no conflict of interest.

## Supporting information


Appendix S1

Figure S1

Figure S2

Figure S3

Table S1

## Data Availability

Data that support the findings of the present study are available from the corresponding author upon reasonable request.
