# Supplementary material for: Microbial lipopolysaccharide‐induced inflammation contributes to cognitive impairment and white matter lesion progression in diet‐induced obese mice with chronic cerebral hypoperfusion
Source: CNS Neurosci Ther. 2023 Jun 8;29(Suppl 1):200–12. doi: 10.1111/cns.14301 (PMC10314110; doi:10.1111/cns.14301)
Supplement: Supplementary file 1 — Appendix S1 [file CNS-29-200-s003.docx]

**Supplementary Method 1**

- 1. **DNA extraction and 16S rRNA microbiome analysis**

Feces were collected from 12-week-old mice fed either the HFD or LFD, lyophilized using a VD-800R lyophilizer (TAITEC, Saitama, Japan) for at least 18 h, and disrupted using 3.0-mm zirconia beads by vigorous shaking (1,500 rpm for 10 min) on a Shake Master (Biomedical Science, Tokyo, Japan). Samples (10 mg) were suspended in DNA extraction buffer containing 400 μL of 10% (w/v) SDS/TE (10 mM Tris-HCl, 1 mM EDTA, and pH 8.0) solution and 400 μL of phenol/chloroform/isoamyl alcohol (25:24:1), and further disrupted using 0.1-mm zirconia/silica beads by vigorous shaking (1,500 rpm for 5 min) on a Shake Master. After centrifugation at 17,800 ×*g* for 5 min at room temperature, DNA was extracted using an automated system (GENE PREP STAR PI-480, Kurabo Industries Ltd., Osaka, Japan) according to the manufacturer’s protocol. The V1-V2 region of the 16S rRNA gene was amplified using primers 27F-mod (5´-AGRGTTTGATYMTGGCTCAG-3´) and 338R (5´-TGCTGCCTCCCGTAGGAGT-3´), and Tks Gflex DNA polymerase (TaKaRa, Shiga, Japan).^1^ The amplified DNA was sequenced using Miseq (Illumina, San Diego, CA, USA) and v3 reagent (for 300 bp pair-end reads) according to the manufacturer’s protocol.

- 1. **Bioinformatics**

QIIME2 (version 2019.10) was used to analyze the microbiome lineage composition.^2^ Briefly, the primer sequence was removed with cutadapt,^3^ followed by preprocessing for quality filtering and denoising using DADA2 (options: --p-trunc-len-f 230 --p-trunc-len-r 130).^4^ The denoised output Amplicon Sequence Variant (ASV) representative sequences were assigned to taxa using the “qiime feature-classifier classify-sklearn” command with default parameters. Silva SSU Ref Nr 99 (version 132) was used as a reference database for taxonomy assignment.^5^ Alpha diversity was calculated using observed ASVs, the Shannon index, and Faith’s phylogenetic diversity. Alpha diversity and fluctuations of individual bacteria were compared between the HFD and LFD groups by Wilcoxon’s rank-sum test (scipy version 1.5.2). Multidimensional scaling was performed using the weighted UniFrac distance.

**REFERENCES**

1. Kim SW, Suda W, Kim S, Oshima K, Fukuda S, Ohno H, et al. Robustness of gut microbiota of healthy adults in response to probiotic intervention revealed by high-throughput pyrosequencing. *DNA Res.* 2013;20(3):241-253.
2. Bolyen E, Rideout JR, Dillon MR, Bokulich NA, Abnet CC, Al-Ghalith GA, et al. Reproducible, interactive, scalable and extensible microbiome data science using QIIME 2. Nat Biotechnol. 2019;37(8):852-7.
3. Martin, M. Cutadapt removes adapter sequences from high-throughput sequencing reads. EMBnet.journal. 2011;17(1):10-12.
4. Callahan BJ, McMurdie PJ, Rosen MJ, Han AW, Johnson AJ, Holmes SP. DADA2: High-resolution sample inference from Illumina amplicon data. Nat Methods. 2016;13(7):581-3.
5. Quast C, Pruesse E, Yilmaz P, Gerken J, Schweer T, Yarza P, et al. The SILVA ribosomal RNA gene database project: improved data processing and web-based tools. Nucleic Acids Res. 2013;41(Database issue):D590-6.
