# Supplementary figures and images for: Microbial lipopolysaccharide‐induced inflammation contributes to cognitive impairment and white matter lesion progression in diet‐induced obese mice with chronic cerebral hypoperfusion
Source: CNS Neurosci Ther. 2023 Jun 8;29(Suppl 1):200–12. doi: 10.1111/cns.14301 (PMC10314110; doi:10.1111/cns.14301)

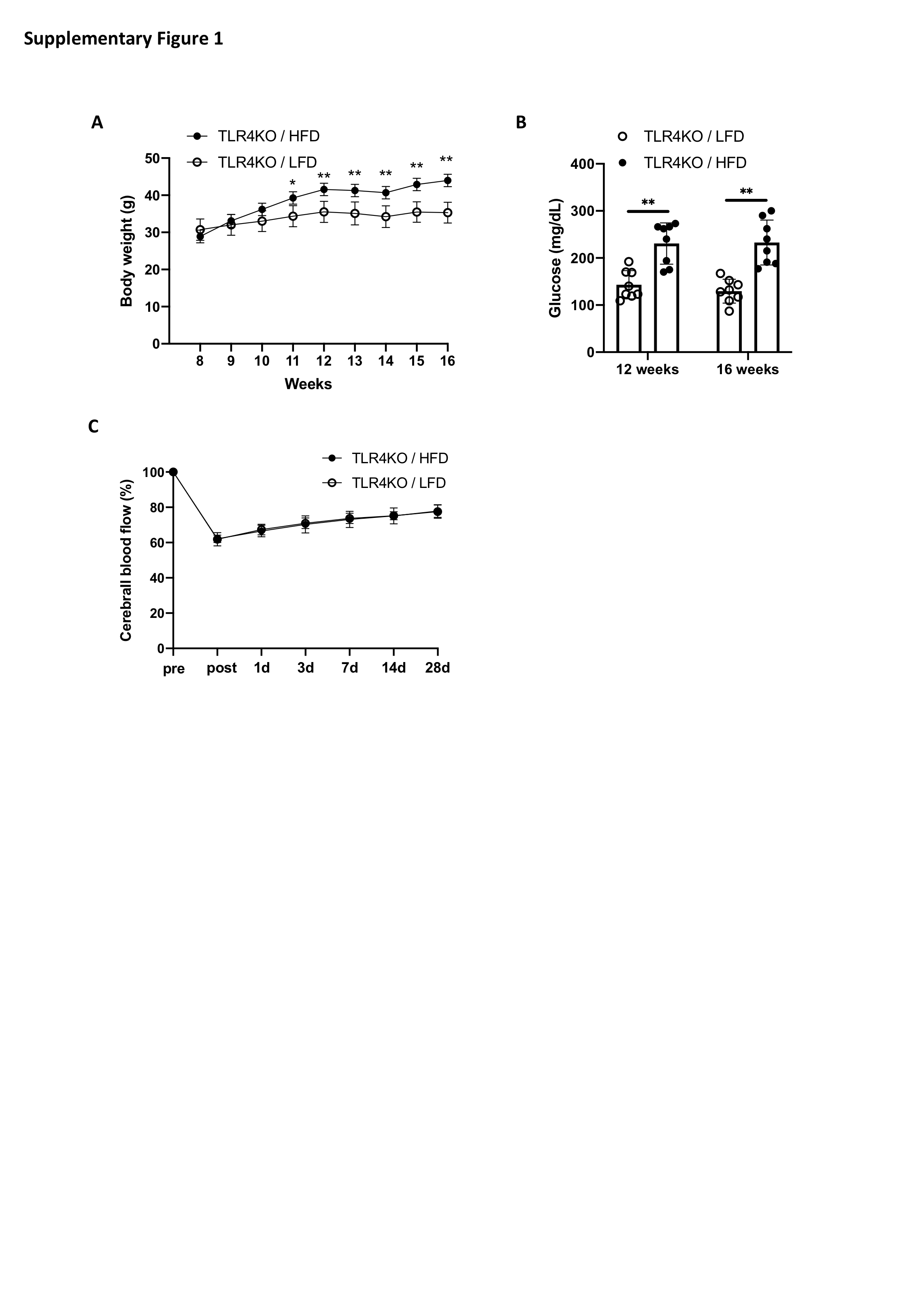

Supplement: Supplementary file 2 — Figure S1 [file CNS-29-200-s001.tiff]

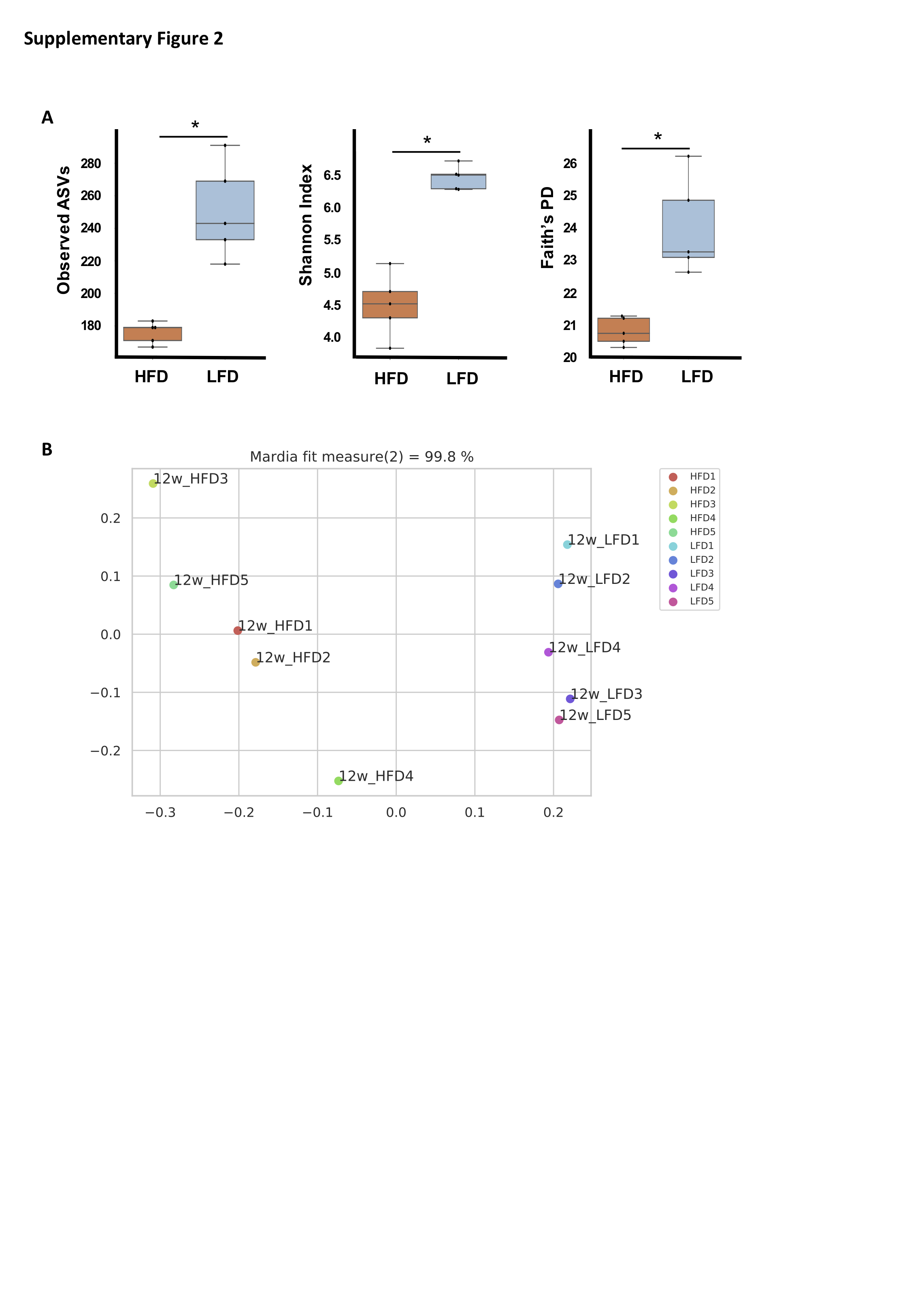

Supplement: Supplementary file 3 — Figure S2 [file CNS-29-200-s005.tiff]

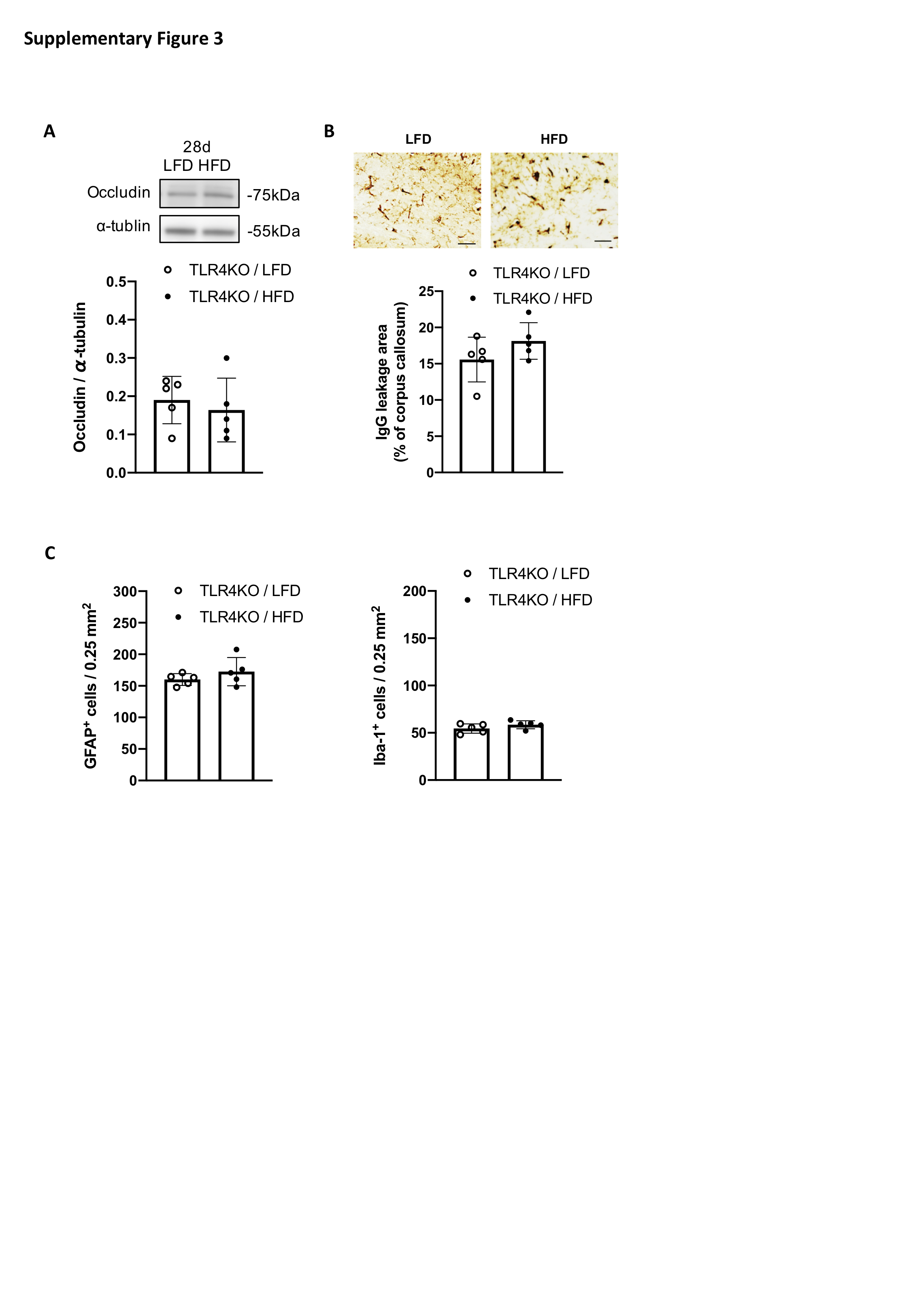

Supplement: Supplementary file 4 — Figure S3 [file CNS-29-200-s002.tiff]
