# Supplementary material for: Microbial lipopolysaccharide‐induced inflammation contributes to cognitive impairment and white matter lesion progression in diet‐induced obese mice with chronic cerebral hypoperfusion
Source: CNS Neurosci Ther. 2023 Jun 8;29(Suppl 1):200–12. doi: 10.1111/cns.14301 (PMC10314110; doi:10.1111/cns.14301)
Supplement: Supplementary file 5 — Table S1 [file CNS-29-200-s004.docx]

**Supplementary Table 1.** Composition of High-Fat Diet 32 and the Low-Fat Diet

| Ingredient, % | High Fat Diet 32 | Low Fat Diet |
| --- | --- | --- |
| Casein | 24.5 | 17.5 |
| Albumen | 5.0 | 3.6 |
| L-Cystine | 0.43 | 0.3 |
| Beef tallow | 15.88 | 1.95 |
| Safflower oil | 20.0 | 2.45 |
| Corn Starch | 0 | 51.1486 |
| Cellulose | 5.5 | 4.0 |
| Maltodextrin | 8.25 | 2.0 |
| Lactose | 6.928 | 2.3 |
| Sucrose | 6.75 | 0 |
| Granulated sugar | 0 | 10.0 |
| Vitamin Mix, AIN93 | 1.4 | 1.0 |
| Mineral Mix, AIN93G | 5.0 | 3.5 |
| Choline Bitartrate | 0.36 | 0.25 |
| Tertiary butylhydroquinone | 0.002 | 0.0014 |
| Total | 100.0 | 100.0 |
